# A Computational Modeling and Simulation Approach to Investigate Mechanisms of Subcellular cAMP Compartmentation

**DOI:** 10.1371/journal.pcbi.1005005

**Published:** 2016-07-13

**Authors:** Pei-Chi Yang, Britton W. Boras, Mao-Tsuen Jeng, Steffen S. Docken, Timothy J. Lewis, Andrew D. McCulloch, Robert D. Harvey, Colleen E. Clancy

**Affiliations:** 1 Department of Pharmacology, University of California Davis, Davis, California, United States of America; 2 Department of Biomedical Engineering, University of California San Diego, La Jolla, California, United States of America; 3 Department of Mathematics, University of California Davis, Davis, California, United States of America; 4 Department of Pharmacology, Center for Molecular Medicine, School of Medicine, University of Nevada Reno, Reno, Nevada, United States of America; University of South Alabama, UNITED STATES

## Abstract

Subcellular compartmentation of the ubiquitous second messenger cAMP has been widely proposed as a mechanism to explain unique receptor-dependent functional responses. How exactly compartmentation is achieved, however, has remained a mystery for more than 40 years. In this study, we developed computational and mathematical models to represent a subcellular sarcomeric space in a cardiac myocyte with varying detail. We then used these models to predict the contributions of various mechanisms that establish subcellular cAMP microdomains. We used the models to test the hypothesis that phosphodiesterases act as functional barriers to diffusion, creating discrete cAMP signaling domains. We also used the models to predict the effect of a range of experimentally measured diffusion rates on cAMP compartmentation. Finally, we modeled the anatomical structures in a cardiac myocyte diad, to predict the effects of anatomical diffusion barriers on cAMP compartmentation. When we incorporated experimentally informed model parameters to reconstruct an in silico subcellular sarcomeric space with spatially distinct cAMP production sites linked to caveloar domains, the models predict that under realistic conditions phosphodiesterases alone were insufficient to generate significant cAMP gradients. This prediction persisted even when combined with slow cAMP diffusion. When we additionally considered the effects of anatomic barriers to diffusion that are expected in the cardiac myocyte dyadic space, cAMP compartmentation did occur, but only when diffusion was slow. Our model simulations suggest that additional mechanisms likely contribute to cAMP gradients occurring in submicroscopic domains. The difference between the physiological and pathological effects resulting from the production of cAMP may be a function of appropriate compartmentation of cAMP signaling. Therefore, understanding the contribution of factors that are responsible for coordinating the spatial and temporal distribution of cAMP at the subcellular level could be important for developing new strategies for the prevention or treatment of unfavorable responses associated with different disease states.

## Author Summary

Subcellular compartmentation of the ubiquitous second messenger cAMP has been widely proposed as a mechanism to explain how this one signaling molecule produces unique receptor-dependent functional responses. But, how exactly compartmentation occurs, is unknown. This is because there has been no way to measure the regulation and movement of cAMP in cells with intact subcellular structures. In this study, we applied novel computational approaches to predict whether PDE activity alone or in conjunction with restricted diffusion is sufficient to produce cAMP gradients in submicroscopic signaling domains. We also used the models to test the effect of a range of experimentally measured diffusion rates on cAMP compartmentation. Our simulations suggest that PDE activity alone is not sufficient to explain compartmentation, but if diffusion of cAMP is limited by potential factors such as molecular crowding, PKA buffering, and anatomical barriers, then compartmentation is predicted to occur.

## Introduction

For nearly 40 years, subcellular compartmentation has been offered as an explanation for how cAMP, the ubiquitous and diffusible second messenger, can both regulate a multitude of cellular functions and elicit specific and selective responses. Despite widespread recognition of the importance of cAMP compartmentation in tightly controlling local signaling, exactly how compartmentation occurs is still poorly understood. The general definition of compartmentation in this context is when a gradient exists in the concentration of cAMP between two locations. As it relates to cell signaling, the concentration gradient is relevant when it affects the potential for cAMP to activate an effector, such as protein kinase A (PKA), in one location but not another. A number of processes have been suggested to contribute to this phenomenon, but studies have offered conflicting data that differ in their interpretation and assessment of key players.

Localized degradation by phosphodiesterases (PDEs) has been a prime focus of many studies attempting to understand the basis of cAMP compartmentation [1–5]. Phosphodiesterases are thought to contribute to the generation of cytosolic cAMP gradients either by acting as functional barriers to diffusion that result in lower levels of cAMP distal to its site of production or as sinks that deplete cAMP in localized areas. Evidence clearly demonstrates that PDE activity is an essential factor in cAMP compartmentation. This has been illustrated by employing a number of different experimental approaches, including Jurevcius and Fischmeister who used patch clamp electrophysiology to demonstrate that in frog ventricular myocytes, inhibition of PDE activity allows local stimulation of cAMP by β-adrenergic receptors to enhance distal Ca^2+^ channel activity [6]. On the other hand, Zaccolo et al. used a genetically encoded FRET-based biosensor to demonstrate that β adrenergic stimulation elicits a localized pattern of cAMP production in neonatal cardiac myocytes that is disrupted by inhibition of PDE activity [7]. However, the question of whether or not PDE activity alone is sufficient to explain the compartmentalized behavior of cAMP signaling is still debated.

Computational modeling has proven to be a useful tool in investigating the relative contribution of PDEs to cAMP compartmentation [8]. All modeling studies support the idea that PDE activity is necessary for cAMP compartmentation. At least one study has predicted that it is theoretically possible for artificially high levels of PDE activity alone to explain compartmentation [9]. Other models using more realistic levels of PDE activity suggest that factors such as the shape of the cell and the rate of cAMP diffusion play critical roles in explaining the existence of cAMP gradients within a cell [10–21].

One way that cell shape may be a factor in compartmentalizing cAMP signaling is by affecting the surface-to-volume ratio. Studies using FRET-based biosensors in neurons have found cAMP levels to be higher in dendrites than cell bodies[22]. It was suggested that this could be due to the higher surface-to-volume ratio found in dendrites, resulting in greater membrane bound adenylyl cyclase activity and reduced cytosolic PDE activity. Subsequent modeling supported the feasibility of this hypothesis, without having to assume the involvement of other factors [18]. Feinstein et al. found that the surface-to-volume ratio of a cell can contribute to generation of cAMP gradients, but it was also necessary to assume that the movement of cAMP is slower than the rate of free diffusion [20]. Other models have been able to explain compartmentation independent of cell morphology, as long as it was assumed that cAMP diffusion is somehow restricted [10, 11, 13–15, 17, 23].

Although the potential effect that the surface-to-volume ratio of a cell has on cAMP compartmentation has been examined, the influence of the actual size and shape of subcellular compartments is less well understood. A major reason is that the physical nature of these microdomains is not well described. Previous modeling studies often circumvented this issue by using loosely defined membrane and cytosolic domains and treating the movement of cAMP between them as fluxes that do not require knowledge of the number, size, or location of these compartments.

Previous experimental studies have shown that receptors associated with cholesterol rich lipid rafts, which include caveolae, can elicit cAMP responses that are distinctly different from those produced by extracaveolar receptors found outside of lipid rafts [16, 24, 25]. Lipid rafts are liquid-ordered domains of the membrane rich in cholesterol and sphingolipids. Caveolae are a specific subset of lipid rafts that contain caveolins, proteins involved in the formation of signaling complexes that include β_1_ and β_2_ adrenergic receptors (βARs) as well as adenylyl cyclase isoforms 5 and 6 (AC5/6) [26–29]. In cardiac myocytes, activation of receptors associated with caveolar lipid rafts are involved in local cAMP production and PKA-dependent regulation of L-type Ca^2+^ channel function [24, 28, 29]. There is evidence that these types of compartmentalized cAMP responses also occur in the transverse tubules (t-tubules) of cardiac myocytes[30]. T-tubules are invaginations of the plasma membrane that come in close proximity to the junctional sarcoplasmic reticulum (SR) forming dyadic junctions [31]. Therefore, it is possible that the size, shape, and distribution of caveolae, especially those associated with the restricted space at cardiac dyadic junctions, may contribute to compartmentation of cAMP signaling in cardiac myocytes.

The purpose of the present study was to apply novel computational approaches to predict whether PDE activity alone or in conjunction with restricted diffusion is sufficient to produce cAMP gradients in submicroscopic signaling domains.

## Results

Experimental studies in cardiac myocytes have shown that activation of βARs associated with caveolar regions of the plasma membrane produce unique compartmentalized cAMP responses [24, 25, 28, 29, 32]. Other studies have used computational approaches to investigate the effect that cell morphology has on the generation of cytosolic cAMP gradients [18, 20, 23]. However, the importance that the organization and structure of submicroscopic signaling domains has on creating compartmentalized cAMP responses has not been addressed. To investigate how cAMP-mediated responses are localized and prevent initiation of global responses, we developed an idealized, partial differential equation (partialDE)-based 2D continuum model of a cardiac myocyte subspace with spatially distinct cAMP microdomains to allow for simulation of cAMP compartmentation and diffusion.

**Fig 1:**
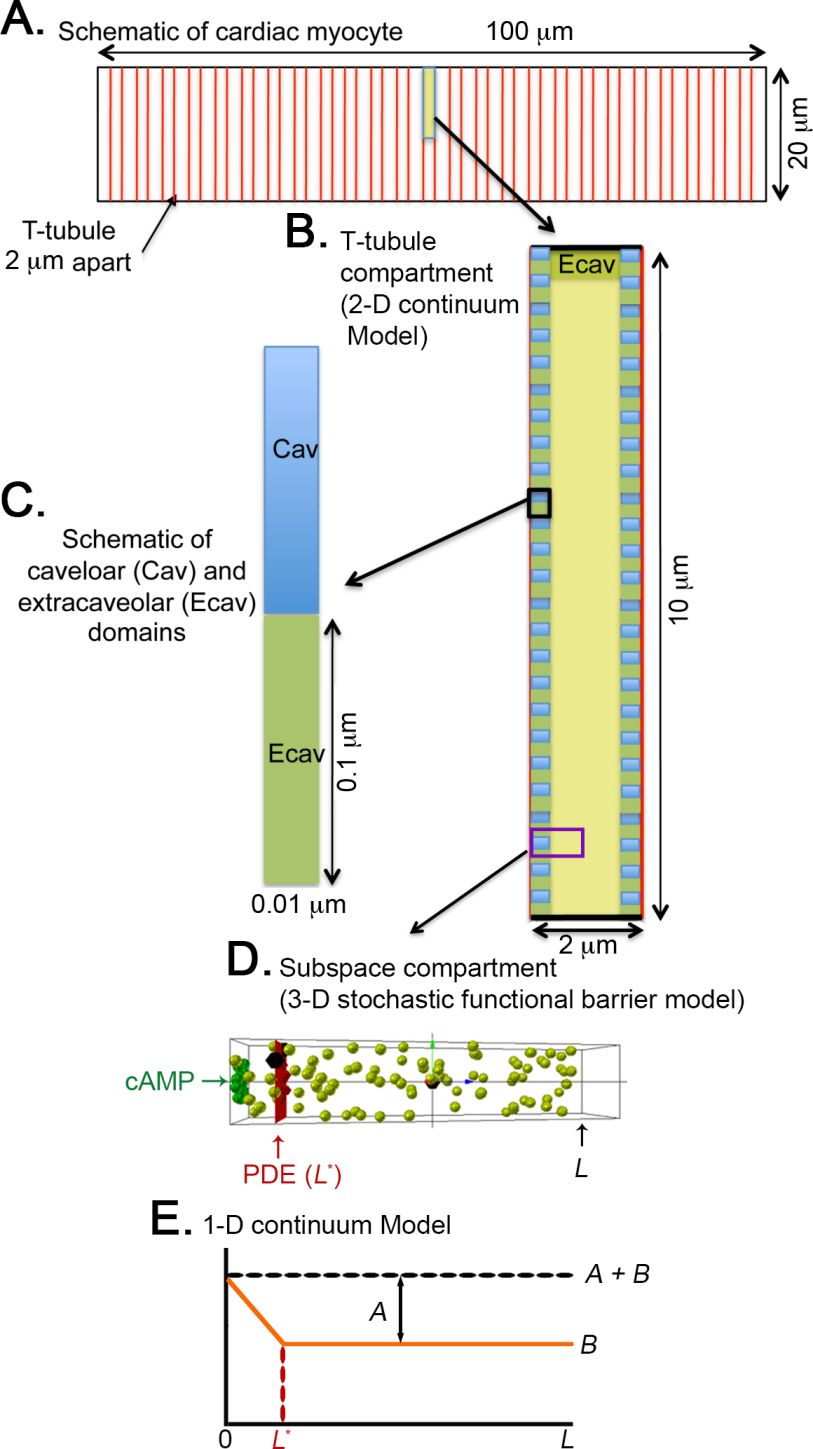
Schematic illustration of the construction of the partialDE models and Mcell stochastic simulations for cAMP compartmentation and diffusion. (A) The longitudinal cross-section of an adult ventricular myocyte, which is 100 μm x 20 μm. (B) The repeating pattern of the intracellular space between adjacent t-tubules, which are 2 μm apart. Each unit is half the width of the cell (10 μm). The 2-D continuum model is at this level. (C) The sarcolemmal membrane lining the t-tubules contains caveolar domains ^51^ where βARs and AC5/6 are localized. Caveolar domains (blue) are 100 nm x 10 nm, spaced 100 nm apart. In this example, extracaveolar domains (green) are associated with the subsarcolemmal space of the t-tubules (between caveolar domains) as well as the peripheral sarcolemma. (D) A single caveolar domain and half of each adjacent extracaveolar flanking region. All of the PDE modelcules are located at a distance (L*) from the plasma membrane (the site of cAMP production). *L* indicates the most distal site from the plasma membrane in the compartment. The Mcell simulations were carried out in a subspace compartment (from L* to *L*) on this microdomain. (E) A schematic of the steady state distribution of cAMP along the microdomain in D as derived using the 1-dimensional continuum model. The concentration is *A+B* at the cAMP production site (z = 0). Beyond the PDE barrier (from L* to *L*), the concentration of cAMP is *B*.

**Fig 2:**
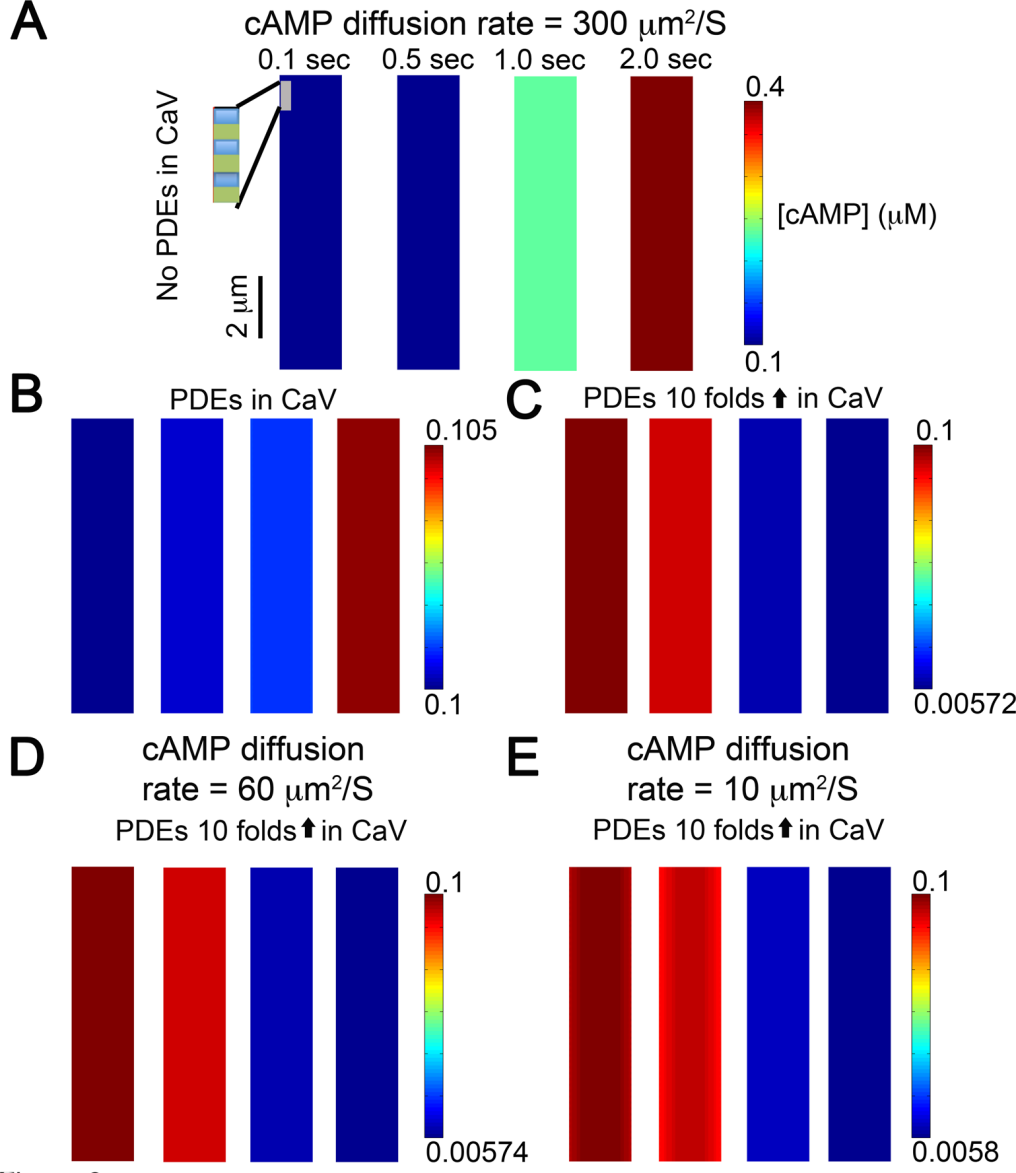
Idealized partialDE model demonstrating cAMP generation and diffusion (diffusion coefficient 300 μm^2^/s) from t-tubular caveolar microdomains (seen as rectangles along edge of inset) at various time points following β_1_AR stimulation with 30 nM isoproterenol (basal cAMP = 0.1 μM). (A) As expected, in the absence of PDEs or anatomical barriers, diffusion is rapid with miniscule gradients and cAMP concentration grows unboundedly. (B) To simulate effects of PDEs, experimentally measured concentrations of PDEs were added into caveolar microdomains (10 PDE molecules/cavelor domain), and (C) Effect of 10-fold increase in the concentration of PDEs. No gradients were observed. (D) When diffusion coefficient was set to 60 μm^2^/s, the simulated results showed sub-nanomolar gradients before 1.0 second. (E) Diffusion coefficient was set to 10 μm^2^/s. Miniscule gradients (sub-nanomolar) were observed during the early time periods (before 1.0 second) when concentration of PDE was increased 10 fold.

A schematic diagram of the longitudinal cross-section of an adult ventricular myocyte with dimensions of 100 μm x 20 μm [14] is shown in Fig 1A. This illustrates the repeating pattern of the sarcomeres, which are spaced 2 μm apart [33]. Fig 1B illustrates the 2D continuum model that we constructed to represent the subcellular sarcomeric space used in the simulations shown in Fig 2. The model represents the intracellular space between adjacent t-tubules. The t-tubules are lined by caveolar domains spaced 100 nm apart [34, 35]. Each unit is half the width of the cell (10 μm). Fig 1C shows a magnified section of the subsarcolemmal space consisting of a single caveolar domain (blue), which is 0.1 μm x 0.01 μm [34, 36, 37], and an adjacent 0.1 μm x 0.01 μm extracaveolar space (green).

β-adrenergic receptors (βARs) and adenylyl cyclases (ACs) (the site of cAMP production) were distributed equally among the 50 caveolar domains on each side of the sarcomeric space. Using this model we would expect to be able to readily track compartmentation as cAMP gradients. We define a “significant” gradient as one in which the concentration of cAMP drops by more than 15% of its value relative to the site of production. Furthermore, we identify gradients as being relevant to compartmentalized signaling if cAMP concentrations in one compartment reach levels likely to produce PKA activation (>1 μM).

Predictions from simulations using this 2D continuum partialDE model to simulate cAMP diffusion are shown as snapshots of cAMP concentration at different points in time across the microdomain space in Fig 2. The time course of spatial changes in cAMP concentration resulting from activation of βARs is shown when cAMP was allowed to move at a rate approximating free-diffusion (300 μm^2^/s) under conditions where no phosphodiesterases (PDEs) were present (Fig 2A), where PDEs were localized to the caveolar domain at concentrations consistent with those reported experimentally [14, 15] (Fig 2B), and where PDE concentrations in the caveolar domain were increased 10-fold (Fig 2C). Only miniscule gradients (sub-nanomolar) were observed during the 2.0 second simulations, even when the concentration of PDE was increased 10-fold. The prediction of the model led to no indicators of significant compartmentation, which we would have expected to observe as gradients of cAMP concentration within the subcellular sarcomeric space. Rather, the monochromatic color maps in Fig 2 at each time point indicates a uniform cAMP concentration.

Several studies [10–17, 20, 23, 38] have suggested that for compartmentation to occur, diffusion of cAMP must be substantially slower than the reported value of free diffusion in a dilute aqueous environment, which is 300 to 400 μm^2^/s [39, 40]. Assuming that cAMP movement is affected by factors such as cytoplasmic viscosity and molecular crowding, the diffusion coefficient of molecules the size of cAMP has been estimated at 60 μm^2^/s [41]. However, slowing diffusion in our simulation was still insufficient to generate a spatial gradient, even when the amount of PDE activity was increased 10-fold above the levels believed to exist in cardiac myocytes (Fig 2D). It has also been suggested that buffering of cAMP through its interactions with PKA can decrease the effective diffusion coefficient for cAMP even further, to values closer to 10 μm^2^/s [41]. Yet, even this marked reduction of cAMP diffusion rate in the simulation was insufficient to generate spatial gradients of cAMP (Fig 2E).

**Fig 3:**
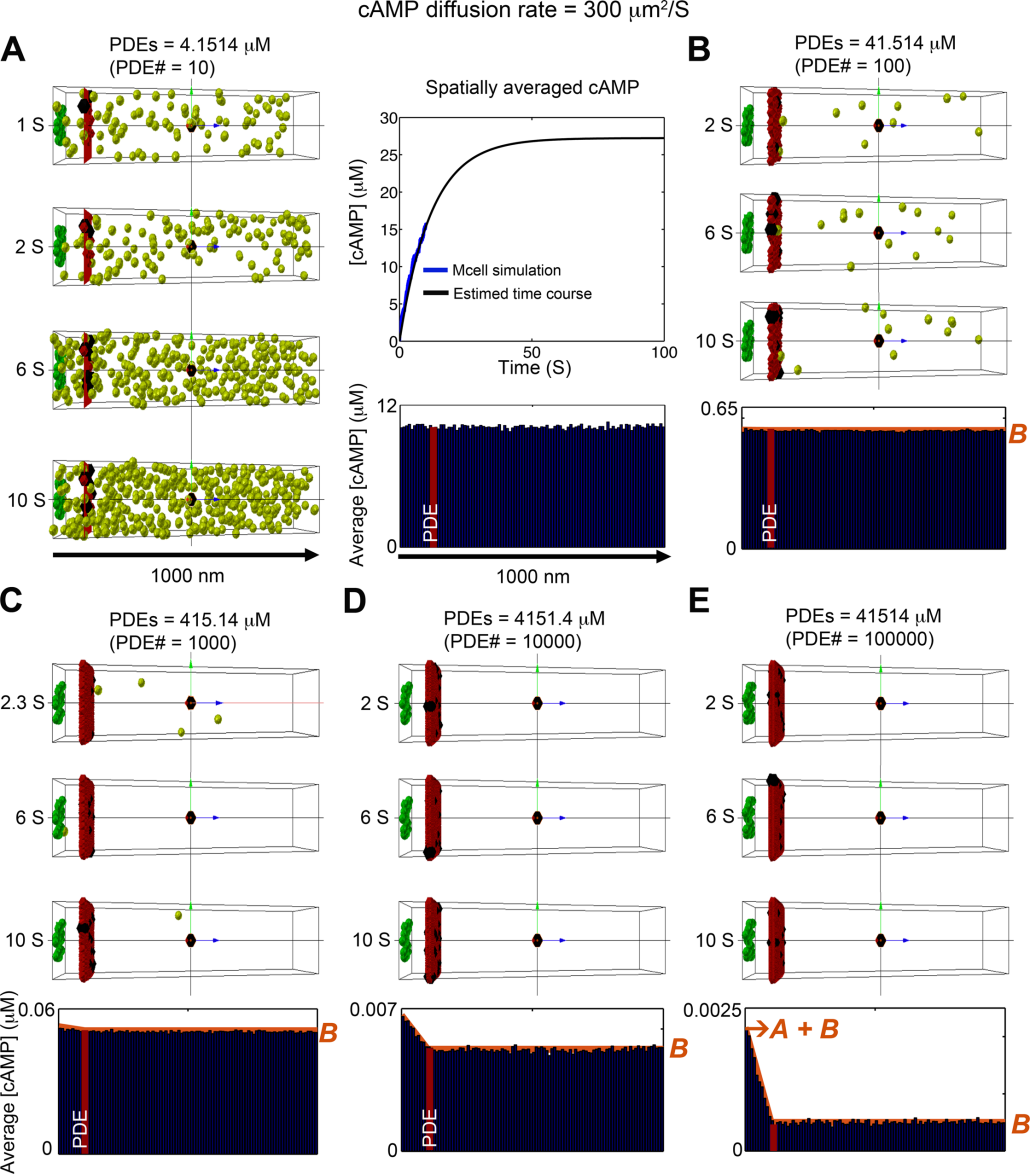
Stochastic simulation of cAMP diffusion implemented in MCell and visualized using CellBlender. Snapshots in time of cAMP distribution generated by a single caveolar domain (green box) containing 15 β_1_ARs, surrounded by non-caveolar space (200 x 200 x 1000 nm), which produced cAMP 120 molecules/s. Freely diffusing cAMP molecules are shown in light green. PDE molecules were placed on a plane at z = 100 nm from the caveloar domain as functional barriers (red plane). (A) 10 PDE molecules (~4.1514 μM). Four time snapshots are shown on the left panels, average concentration of cAMP over 1800 time frames from 1s to 10s are shown in the blue bar graph in the bottom right panel. The top right panel shows the time course of the spatially averaged cAMP concentration over the full domain; simulated data are shown as blue line, and black line depicts an exponential curve that show the approach to steady state. (B-E) The effects of vaying PDE concentration. Three time snapshots are shown in the top panels, and average concentration of cAMP molecules for 1800 time frames from 1s to 10s at steady state are shown in the blue bar graph in the bottom right panels. (B) PDE molecules = 100 (~41.514 μM). (C) PDE molecules = 1000 (~415.14 μM). (D) PDE molecules = 10000 (~4151.4 μM). (E) PDE molecules = 100000 (~41514 μM). The red curves plotted on the accumulated concentration maps in panel (B-E) show the predictions of the 1D continuum model. In all cases, there is excellent agreement with the full 3D stochastic model. The cAMP compartmentation ratio *R* (see text) for the various values of PDE concentration shown in panels (A-E) are 6.098 x 10^−5^, 3.040 x 10^−3^, 3.188 x 10^−2^, 2.491 x 10^−1^, and 7.685 x 10^−1^.

**Fig 4:**
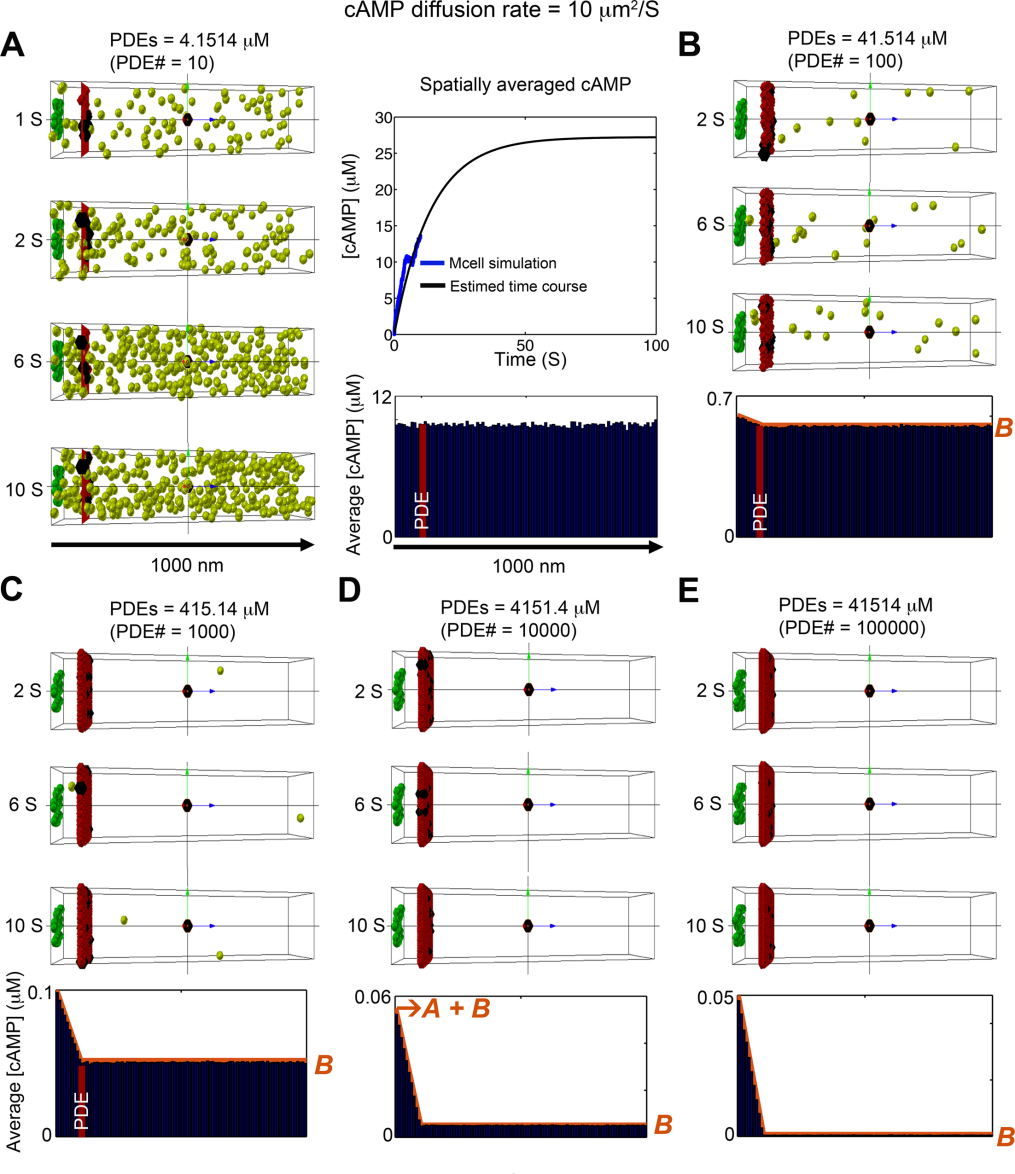
Stochastic simulation of cAMP diffusion implemented in MCell and visualized using CellBlender. The diffusion coefficient was set to ***10 μm***^***2***^***/s***. (A) 10 PDE molecules (~4.1514 μM). Four snapshots are shown in the left, the average cAMP concentration for the 1800 time frames between 1s to 10s are shown shown in the blue bar graph, and the time course of the spatially averaged cAMP concentration is shown in the top right panel. (B) PDE molecules = 100 (~41.514 μM). Average of cAMP molecules for 1800 time frames from 1s to 10s at steady state. (C) PDE molecules = 1000 (~415.14 μM). Average of cAMP molecules over 1800 time frames from 1s to 10s at steady state. (D) PDE molecules = 10000 (~4151.4 μM). Average of cAMP molecules for 1800 time frames from 1s to 10s at steady state. (E) PDE molecules = 100000 (~41514 μM). Average of cAMP molecules over 1800 time frames from 1s to 10s at steady state. The red curves plotted on the accumulated concentration maps in panel (B-E) show the predictions of the 1D continuum model. In all cases, there is excellent agreement with the full 3D stochastic model. The cAMP compartmentation ratio *R* for the various values of PDE concentration shown in panels (A-E) are 1.826 x 10^−3^, 8.381 x 10^−2^, 4.970 x 10^−1^, 9.087 x 10^−1^, and 9.901 x 10^−1^,

In the 2D continuum model, PDEs were contained within the thin caveloar domain (i.e. all PDE is effectively along the plasma membrane). In order to more specifically address the question of whether PDEs can form a “functional barrier” to cAMP diffusion, we developed a 3D stochastic model of cAMP diffusion in a subcellular microdomain (depicted in Fig 1D) and implemented the model using MCell [42]. This approach allows for investigation of the contribution of spatial localization of microdomain specific signaling components (e.g., PDEs) to compartmentation. Evidence exists that the localization of signaling complexes is important in producing compartmentalized responses [28, 43]. Shown in Fig 3 are results from stochastic simulations of cAMP diffusion visualized using CellBlender (mcell.org). Fig 1D illustrates the subcellular compartment model that is used in the 3D stochastic simulations described in Fig 3 through Fig 4. The space (200 x 200 x 1000 nm) surrounding a single caveolar domain containing 15 βARs is depicted in green in Fig 3A–3E. Activation of βARs generated 120 cAMP molecules/s. Once produced the cAMP molecules diffused freely in the microdomain at a rate of 300 μm^2^/s. PDE molecules were placed in a plane (L*) 100 nm from the inner surface of the plasma membrane (red plane in Fig 3A–3E) in order to determine if PDEs could act as a functional barrier to cAMP diffusion.

Fig 3A illustrates the distribution of cAMP molecules throughout the microdomain at various time points when the functional barrier consists of 10 PDE molecules. This corresponds to a PDE concentration of 4.15 μM. The graph at the top right of Fig 3A illustrates the time course of cAMP accumulation under these conditions. The graph at the bottom right of Fig 3A plots the accumulated concentration of cAMP (averaged over the first 1 to 10 second time interval along the length of the microdomain. The simulation demonstrates that 10 PDE molecules are not enough to serve as a functional barrier to cAMP diffusion and generate a discernible cAMP gradient.

We then evaluated the effect of increasing the number of PDE molecules in the functional barrier by several orders of magnitude (Fig 3B–3E). Only when the number of PDE molecules was increased above 10,000 (Fig 3D and 3E) did a cAMP gradient become visible. This is illustrated most clearly by the accumulated concentration map at the bottom of each panel.

The results described above indicate that PDE activity alone is unlikely to produce significant cAMP gradients by acting as a functional barrier when cAMP was allowed to diffuse freely. We next tested if this was also the case when the rate of cAMP diffusion was decreased to 60 μm^2^/s, as shown in S3 Fig. This condition reflects the experimentally measured diffusion coefficient of cAMP like molecules that was determined by using fluorescein and the **ϕ**450 fluorophore, fluorescent molecules about the same size as cAMP that do not bind to PKA. In water, these molecules exhibit rates of free diffusion of ~300 μm^2^/s, but inside cardiac myocytes the diffusion coefficient decreases to ~60 μm^2^/s, attributable to collision with other macromolecules in the intracellular environment due to molecular crowding [44]. Despite the slower rate of diffusion, a functional barrier consisting of 10 PDE molecules was still not sufficient to produce a cAMP gradient (S3 Fig). In the setting of slower diffusion, it was necessary to increase the number of PDE molecules to at least 1000 (S3C–S3E Fig) before a small gradient was visible. Slowing the rate of cAMP diffusion also increased the concentration of cAMP observed at all levels of PDE activity.

We then repeated the simulations using a diffusion coefficient of 10 μm^2^/s (Fig 4). This reflects the further slowing of cAMP diffusion due to the effects PKA buffering as suggested experimentally [41]. Interestingly, under these conditions, there is evidence for cAMP compartmentation when the number of PDE molecules in the barrier is at least 100, which corresponds to a concentration of 41.5 μM (Fig 4B–4E).

Even with a diffusion coefficient of 10 μm^2^/s, the diffusion length (2$\sqrt{Dt}$) of cAMP on relevant time scales (1–10 seconds) is much larger than the length scale of the caveolar domain (0.01–0.2μm). This leads to a nearly uniform concentration of cAMP in planes parallel to the plasma membrane, and therefore cAMP dynamics can be well-predicted by a 1D continuum model that can be solved analytically to obtain an expression for the steady state concentration of cAMP along the microdomain (see Methods and S1 Appendix). (Fig 1E shows a schematic of the steady state distribution of cAMP.) The concentration decreases linearly from a value of *A+B* at the cAMP production site (z = 0) to a value of *B* at the location (L*) of the PDE molecules that form a barrier to cAMP. Beyond the PDE barrier (from L* to *L*), the concentration of cAMP is constant at *B*. The orange curves plotted on the cumulative concentration maps in Figs 3 and 4 show the predictions of the 1D continuum model. In all cases, there is excellent agreement with the full 3D stochastic model.

Note that the ratio *R = A/(A+B)* provides a measure of compartmentation: *R = 0* implies that there is a uniform distribution of cAMP throughout the cytosol (no compartmentation), whereas *R = 1* implies that all cAMP is trapped behind the functional barrier of PDEs (complete compartmentation). The cAMP compartmentation ratio *R* for all cases shown in Figs 3 and 4 are provided in the figure captions.

**Fig 5:**
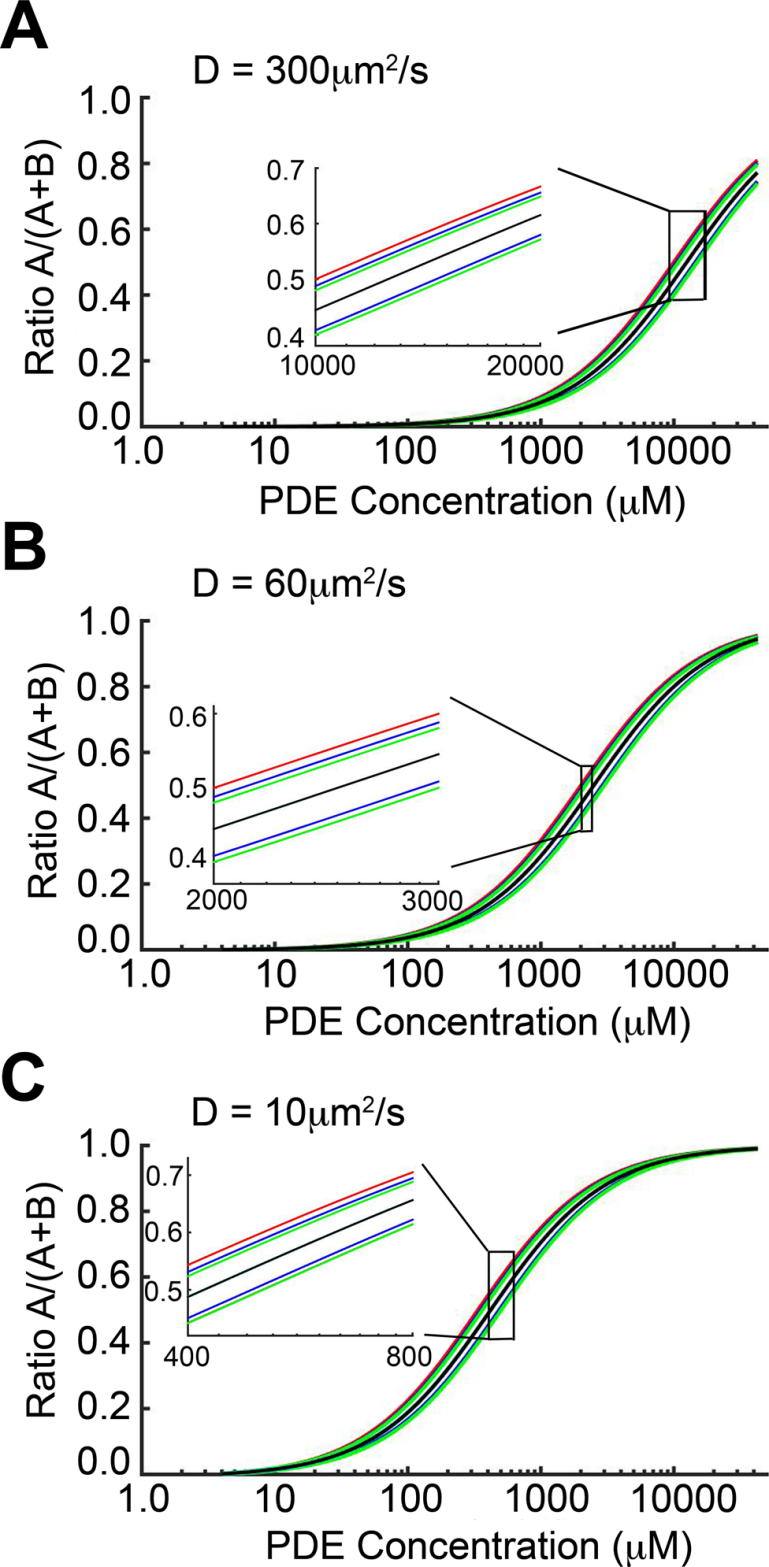
Sensitivity analysis using 1-dimensional continuum model. cAMP compartmentation ratio *R* as a function of PDE concentration for diffusion constants of (A) 300, (B) 60, and (C) 10 μm^2^/s, respectively. Black lines correspond to default parameter values used in Figs 3, 4, and 5. Pairs of colored lines show *R* when the parameters D/(L*k_*f*_) (red), *k*_*b*_ (blue), *k*_*cat*_ (green), and *J*_*B*_ (cyan) were adjusted by ±20%. Note that, in all cases, the lower red line is obscured by a green line and both cyan lines are almost entirely obscured by the black line. For PDE concentrations between 1 and 100 μM, *R* was insensitive to perturbations to parameters (in terms of change of the absolute magnitude of the ratio).

Thus far, our modeling results suggest that under physiologically relevant conditions, cAMP diffusion is not sufficiently restricted by the presence of PDE molecules to explain compartmentation. To further test the effects of the model parameter values on cAMP compartmentation, we used the 1D continuum model to perform a parameter sensitivity analysis. The black curves in Fig 5A shows the dependence of the cAMP compartmentation ratio *R* on PDE concentration for default parameters and a diffusion rate of 300 μm^2^/s as used in Fig 3. The red, blue, green, and cyan lines illustrate the sensitivity of *R* to changes in *D/(k*_*f*_ L*) (a parameter accounting for diffusion, the location of the PDE boundary, and the rate of cAMP association with PDE), *k*_*b*_ (rate of cAMP dissociation from PDE), *k*_*cat*_ (PDE catalysis rate), and *J*_*B*_ (cAMP production rate), respectively. Each parameter was adjusted by ±20%, and the results are plotted as a pair of colored lines for each parameter change. Fig 5B and 5C show the sensitivity to the parameters for cAMP diffusion constants of 60 and 10 μm^2^/s, respectively, as in S3 Fig and Fig 4. For all cases, the cyan lines are almost entirely obscured by the black lines, indicating that the cAMP compartmentation is insensitive to changes in the cAMP production rate, *J*_*B*_. The compartmentation ratio *R* was most sensitive to *D/(k*_*f*_ L*) and *k*_*cat*_, however no change in *R* greater than 0.06 was observed. It is notable that the highest sensitivity of *R* (in terms of change of the absolute magnitude of the ratio) occurred in the ranges of PDE concentrations that are well above the physiologically relevant range. For PDE concentrations between 1 and 100 μM, *R* was insensitive to perturbations to all other parameters.

**Fig 6:**
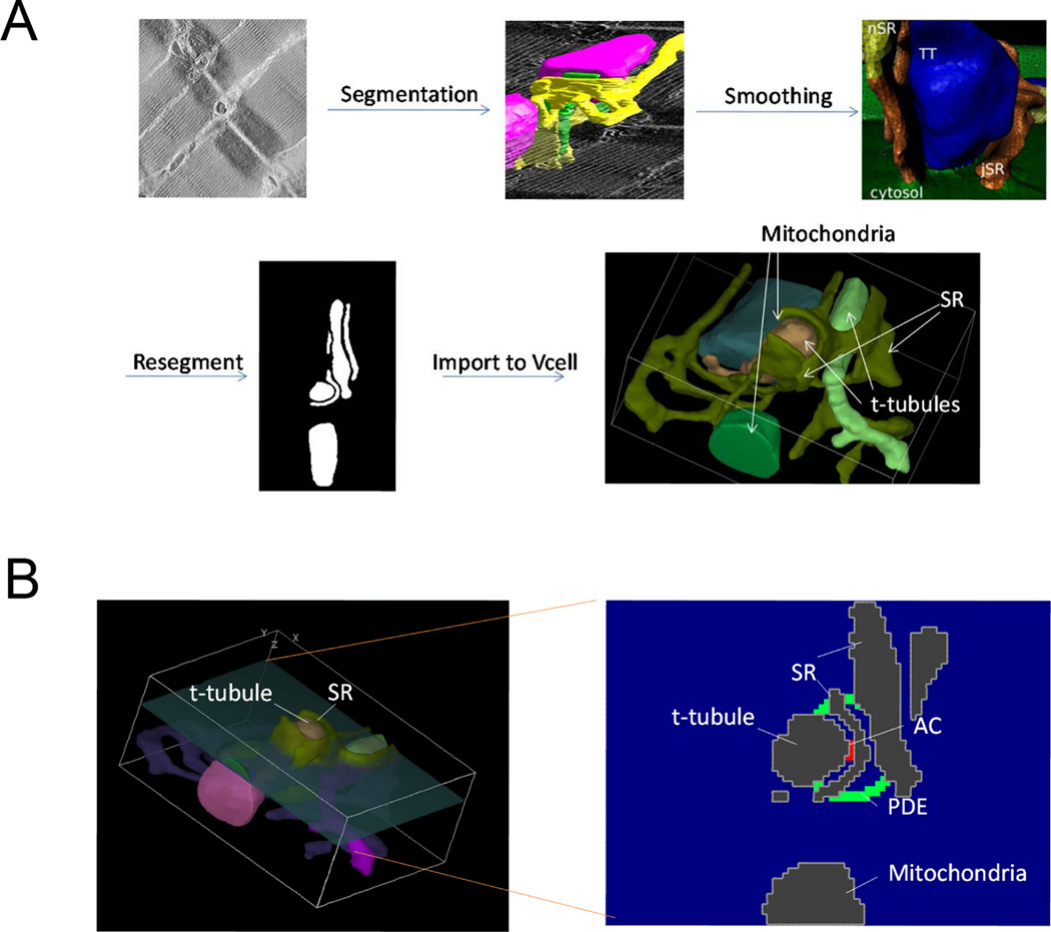
A 3-dimensional continuum anatomical barrier model. **A)** A cryo-TEM z-stack is segmented to create an initial geometry. This is then imported into Blender and smoothed using Blamer. The resultant surface mesh is resegmented and imported into Virtual Cell. **B) Left:** 3D view of the geometry showing the cross section of the geometry where the PDEs were located. **Right:** Cross-sectional view of the placement of the 25 nm radius sphere of PDEs on the inside of the cleft between the t-tubule and the SR.

**Fig 7:**
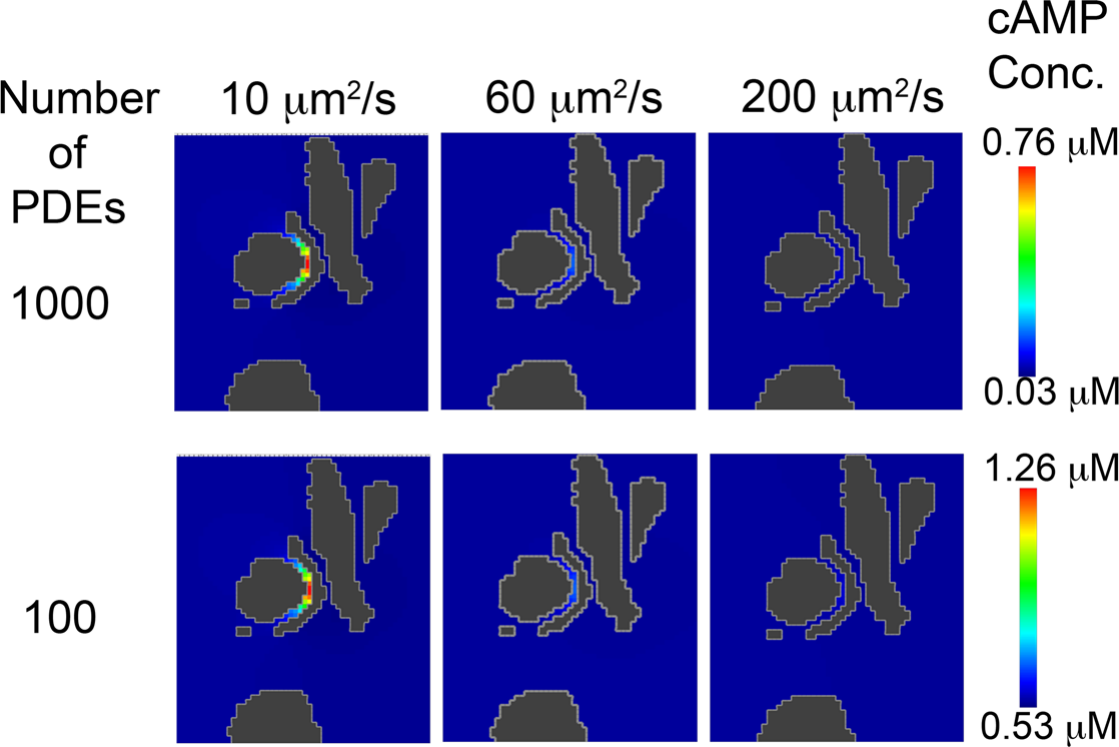
Effect of cAMP diffusion coefficient and PDE concentration in the setting of an anatomical barrier on cAMP compartmentation. 1000 and 100 PDE molecules were placed in a hollow sphere surrounding 15 AC molecules. A cAMP diffusion coefficient of 10 μm^2^/s shows substantial compartmentation, whereas 60 μm^2^/s and 200 μm^2^/s produced minimal cAMP gradients.

The simulations conducted thus far used models incorporating an idealized view of the 3D space between t-tubules in a cardiac myocyte. None of them contained realistic subcellular structures that might, in an actual cell, act as physical barriers to diffusion of cAMP. The cytosolic compartment of a cardiac myocyte is structurally complex and the site of cAMP production in t-tubules likely occurs in close proximity to the junctional SR, forming dyadic clefts. Movement of cAMP out of this space is also likely to be affected by the presence of mitochondria, which make up approximately 30% of the cardiac myocytes volume and are tightly packed around these structures. To examine the possibility that cAMP compartmentation might be observed in this type of restricted space, we created a 3D continuum anatomical barrier model using cryo-TEM images of adult mouse cardiac myocytes, as described previously[45] (Fig 6A). The dimensions of the resulting dyadic cleft were approximately 1040 x 765 x 415 nm. Production of cAMP was generated by 15 AC molecules situated in the center of the dyadic cleft. These were surrounded by a hollow sphere of PDEs 25 nm thick and 200 nm in diameter. The t-tubules, SR, and mitochondria were assumed to be impenetrable barriers to direct diffusion of cAMP throughout the cytosol. We then examined the effects of varying PDE activity, as well as the diffusion coefficient, on cAMP gradients (Fig 7).

When cAMP was produced at the same rate as in the previous simulations (120 molecules/s), and it was allowed to diffuse at a rate of 200 μm^2^/s, which is consistent with previous estimates of the diffusion coefficient in intact cells [22, 46, 47], no evidence of a gradient was observed when the number of PDE molecules surrounding the site of production was varied between 100 and 1000 (Fig 7). This behavior did not change when the diffusion coefficient for cAMP was reduced to 60 μm^2^/s. However, when the diffusion coefficient was further reduced to 10 μm^2^/s, a significant gradient was observed at all PDE concentrations.

## Discussion

In this study, we developed several modeling approaches that included discrete cAMP microdomains to carry out simulations to investigate the importance of parameters proposed to be critical to compartmentation, including location and concentration of PDE activity, rates of cAMP diffusion, and anatomical barriers to diffusion.

There is a large body of literature demonstrating that the non-uniform distribution of PDE activity in different subcellular compartments plays a critical role in compartmentation of cAMP dependent responses [5]. This is achieved by various mechanisms, including interactions with A kinase anchoring proteins (AKAPs), which create signaling complexes that include not only PKA, but also adenylyl cyclase [48]. In the present study, we used a 2-dimensional continuum model to evaluate the effect of localizing PDE activity near the site of cAMP production. The results demonstrate that concentration of PDE activity in this location did not prevent cAMP diffusion throughout the space representing the area between adjacent t-tubules.

Furthermore, a 10-fold increase in PDE activity was still insufficient to restrict cAMP diffusion, rather it reduced cAMP levels throughout the entire compartment. In previous modeling studies using similar levels of PDE activity, cAMP gradients could be generated if was assumed that the movement of cAMP was restricted by some mechanism [10, 14, 15, 20, 23, 49]. However, we found that reducing the cAMP free diffusion coefficient to levels similar to those recently described in intact cardiac myocytes [41], did not promote compartmentation. PDEs were also not enough to prevent the experimentally observed higher concentrations of cytoplasmic cAMP from immediately diffusing into the caveloar spaces, where biosensors have suggested 10-fold lower cAMP concentrations under basal conditions (S2 Fig) [14, 15].

An alternative explanation for how PDE activity creates compartmentalized cAMP responses is based on the common supposition that PDEs act as functional barriers preventing cAMP diffusion [1, 5, 50]. To test this hypothesis, we developed a 3D stochastic model of cAMP diffusion. When it was assumed that cAMP moved at rates equal to free diffusion (300 μm^2^/s), cAMP gradients could only be observed when the number of PDE molecules in the barrier was unrealistically high (Fig 3). Furthermore, levels of PDE activity sufficient to produce a gradient resulted in overall cAMP levels that are well below those required for activating cAMP. Decreasing the diffusion coefficient for cAMP reduced the level of PDE activity necessary to produce gradients. However, even with a diffusion coefficient of 10 μm^2^/s, it was still necessary to use artificially high levels of PDE activity, and the overall level of cAMP was still well below that necessary to activate any downstream signaling.

We also implemented a one-dimensional functional barrier model, which was possible because the diffusion length for relevant reaction (cAMP degradation by PDE) timescales is much larger than the length scale of the periodic structure of the caveolar-extracaveloar compartments. The one-dimensional functional barrier model allowed for derivation of an analytical expression for the steady-state spatial distribution of cAMP, which we used to identify the dependence of cAMP distribution on model parameters including diffusion coefficients, position of the functional barrier, concentration of PDEs and reaction kinetics. Beyond showing that there is very little compartmentation of cAMP under physiological conditions, the analytical solution indicates that the model results show very little sensitivity to variations in model parameters, an indication of robustness of the models.

The final set of simulations examined the role that physical barriers to diffusion might play in the generation of cAMP gradients. A 3D continuum model was implemented, which included subcellular structures, which acted to impede the diffusion of cAMP. It has been postulated that different “compartments” of cAMP can be carved out by creating an area of high AC surrounded by PDEs to form a barrier to prevent the cAMP produced by the AC from affecting the rest of the cell. Although the TEM images of cardiac myocytes do not show anything that would allow such a barrier to exist [51, 52], this hypothesis is prominent in the literature [1, 5, 50], and so it is valuable to test its conceptual validity. It is possible that AKAPs might bind a greater proportion of PDEs at the edges of the cleft surrounding the AC molecules, but to date no study has suggested this type of cellular localization. If the PDE activity near the site of production exists in restricted anatomically bounded clefts, this may explain how cAMP levels near the site of production are kept low under basal conditions, preventing activation of PKA by much higher cAMP levels found throughout the rest of the cell [14, 15].

The present study demonstrates that gradients consistent with those expected to result in compartmentalized responses can be produced, but only in anatomically restricted spaces, and the dimensions of those spaces are below the resolution limit of light microscopy. Therefore, one would not expect to be able to directly visualize these compartmentalized responses with techniques currently available. However, results obtained using FRET based biosensors together with the targeted application of agonists using scanning ion conductance microscopy have shown that activation of beta2-receptors produces evidence of cAMP responses localized specifically to t-tubules in adult ventricular myocytes, and that these cAMP responses do not propagate throughout the cell [30]. This is consistent with our modeling results demonstrating that cAMP production occurring in dyadic clefts along the tubules are compartmentalized.

If we assume that PDE concentration of an average cardiac myocyte is ~0.1μM [53, 54] and the volume is 31,400 μm^3^, this means that there are ~1.3 x 10^6^ PDE molecules per cell. If we further assume that there are approximately 13,000 dyadic clefts ([55, 56], 10,000–50,000) per myocyte, and all PDE activity in the cell is concentrated in these clefts, this would mean that there are 100 PDE molecules per cleft. At this concentration, we found no evidence of a cAMP gradient across the PDE barrier in the anatomical model when diffusion was set at 200 μm^2^/s. Even if we assumed that the number of PDE per cleft was 10 fold higher, this did not affect our ability to detect a gradient. However, reducing the cAMP diffusion made a significant difference. With a diffusion coefficient of 10 μm^2^/s, a cAMP gradient was observed at all PDE concentrations tested. The results of these simulations support the conclusion that PDE activity alone is not sufficient to explain compartmentation, but if diffusion of cAMP is limited by factors such as molecular crowding, PKA buffering, and anatomical barriers combined, then compartmentation may occur.

The diffusion coefficient of cAMP was determined by using fluorescein and the **ϕ**450 fluorophore, fluorescent molecules about the same size as cAMP that do not bind to PKA. In water, these molecules exhibit rates of free diffusion of ~300 μm^2^/s, but inside cardiac myocytes the diffusion coefficient decreases to ~60 μm^2^/s. This is consistent with the 4 to 5 fold decrease in mobility typically seen with molecules this size, and it has been attributed primarily to collision with other macromolecules in the intracellular environment due to molecular crowding. It turns out that cytoplasmic viscosity is only believed to be a minor player [44]. PKA can be found in both membrane and soluble cellular fractions of most cells. Our recent data suggest that PKA is targeted specifically to the mitochondrial outer membrane by A kinase anchoring proteins (AKAPs) [41]. Our future studies will be aimed at determining the quantitative effects of this anchoring on limiting the diffusion of cAMP.

It is worth noting that this work does not preclude gradients of cAMP across entire cells or non-steady state gradients. Several studies of cell motility in non-cardiac cells have shown that a cAMP gradient can exist across the cell [57]. Also, several neuronal studies have pointed to cAMP gradients as a major feature in the turning behavior of neuronal growth cones [58, 59]. In these cases, the distances under consideration are significantly larger. Also, these systems have different organization of relevant enzymes; for example, one study showed by TEM imaging that significant clusters of AC molecules localize to the synapse in rat neurons [60]. However, these computational experiments show that having 10,000 steep gradients around each cleft or each caveolae is infeasible and suggest that another explanation for the observed compartmentalized nature of PKA activity must be considered.

In this study, we focused on the example of the dyadic cleft as a restricted space that would be expected to affect the generation of cAMP gradients and compartmentalized responses. Restricted spaces created by other means would be expected to have the same effect. For example, cultured neonatal cardiac myocytes may not have dyadic clefts, but they are flatter, which together with the tight packing of mitochondria beneath the plasma membrane may be another way of creating restricted spaces that contribute to compartmentation. It is also likely that factors yet to be identified contribute to compartmentalized responses in cardiac myocytes as well as other cell types.

## Methods

### 2-dimensional continuum model

We constructed a 2 μm by 10 μm two-dimensional finite difference model representing the sarcomeric space between adjacent t-tubules of an adult ventricular myocyte (see Fig 1). Cytosolic domains associated with caveolae found in the plasma membrane of the t-tubules were modeled as 0.1 μm x 0.01 μm spaces. These caveolar domains were flanked on each side by 0.1 μm extracaveolar spaces. βARs and AC5/6 were placed in the plasma membrane associated with caveolar domains.

All the simulations were encoded in C and run on 48-Core AMD Opteron Processors. The implicit numerical method was used to integrate *Eqs 1 & 2*. All parameters used in the model can be found in Iancu-Harvey model [61, 62]. The time step (∆t) was set to 0.001 s. Numerical results were visualized using MATLAB R2014a by The Math Works, Inc.

The concentration of cAMP in each compartment was calculated using the following equations:

### Caveolar domain

G-protein activation module:

$$RG_{S}=\frac{\left(R_{\beta 1free}\times G_{S_{free}}\right)}{G_{S_{free}}+K_{C}}$$ (1)

$$LR_{\beta 1}=\frac{L_{iso}\times \left(R_{\beta 1free}-RG_{S}\right)}{L_{iso}+K_{L}}$$ (2)

$$LRG_{S}=\frac{LR_{\beta 1}\times \left(G_{S_{free}}-RG_{S}\right)}{\left(G_{S_{free}}-RG_{S}\right)+\left(\frac{K_{C}\times K_{H}}{K_{L}}\right)}+\frac{L_{iso}\times RG_{S}}{L_{iso}+K_{H}}$$ (3)

$$R_{\beta 1Total}=R_{\beta 1free}+LR_{\beta 1}+LRG_{S}+RG_{S}$$ (4)

$$\frac{\partial G_{S_{\alpha }GTP}}{\partial t}=LRG_{S}\times k_{act2}+RG_{S}\times k_{act1}-G_{S_{\alpha }GTP}\times k_{hydr}$$ (5)

$$\frac{\partial G_{S_{\beta \gamma }}}{\partial t}=LRG_{S}\times k_{act2}+RG_{S}\times k_{act1}-G_{S_{\alpha }GDP}\times G_{S_{\beta \gamma }}\times k_{reas}$$ (6)

$$\frac{\partial G_{S_{\alpha }GDP}}{\partial t}=G_{S_{\alpha }GTP}\times k_{hydr}-G_{S_{\alpha }GDP}\times G_{S_{\beta \gamma }}\times k_{reas}$$ (7)

$$G_{Total}=G_{S_{free}}+G_{S_{\alpha }GTP}+G_{S_{\alpha }GDP}$$ (8)

cAMP produced by AC5/6:

$$k_{AC5/6}=\left(0.7+\frac{3.8234\times G_{S_{\alpha }GTP}^{0.9787}}{0.1986+G_{S_{\alpha }GTP}^{0.9787}}\right)\times \frac{MW_{AC5/6}}{60}\times 10^{-3}$$ (9)

$$\frac{\partial cAMP_{AC5/6}}{\partial t}=\frac{\left(k_{AC5/6}AC_{5/6}AF_{5/6}\right)ATP}{\text{K}\_\text{mATP}+\text{ATP}}$$ (10)

cAMP degraded by PDEs:

$$\frac{\partial cAMP_{PDEx}}{\partial t}=\frac{\left(k_{PDEx}\times PDEx\right)\times cAMP}{\mathrm{Km}\_\text{PDEx}+\text{cAMP}}$$ (11)

The general formulation used for each PDE isoform (*PDEx*).

cAMP dynamics:

$$\begin{array}{c} \frac{\partial cAMP(x,z,t)}{\partial t}=\frac{\partial cAMP_{AC5/6}}{\partial t}\text{-}\left(\frac{\partial cAMP_{PDE2}}{\partial t}+\frac{\partial cAMP_{PDE3}}{\partial t}+\frac{\partial cAMP_{PDE4}}{\partial t}\right)\\ +D\frac{\partial^{2}cAMP(x,z,t)}{\partial x^{2}}+D_{}\frac{\partial^{2}cAMP(x,z,t)}{\partial z^{2}} \end{array}$$ (12)

### Bulk domain

cAMP dynamics:

$$\frac{\partial cAMP(x,z,t)}{\partial t}=D\frac{\partial^{2}cAMP(x,z,t)}{\partial x^{2}}+D\frac{\partial^{2}cAMP(x,z,t)}{\partial z^{2}}$$ (13)

$${\frac{\partial cAMP}{\partial x}|}_{x=0}=0,\;{\frac{\partial cAMP}{\partial x}|}_{x=WL}=0,\;{\frac{\partial cAMP}{\partial z}|}_{z=0}=0,\;{\frac{\partial cAMP}{\partial z}|}_{z=LL}=0$$

where *D* is diffusion coefficient 300 μm^2^/s (Fig 2A–2C), 60 μm^2^/s (Fig 2D) and 10 μm^2^/s (Fig 2E), and *WL =* 2 μm and *LL* = 10 μm. Definitions and initial values for model parameters were based on experimental data as described in [61, 62] and shown in **Tables 1** and **2**.

**Table 1:** Model parameters from [61, 62].

Parameter	Value	Units	Description
R_β1free_	0.633	μM	Concentration of βAR in Cav compartment
K_H_	0.062	μM	High affinity binding constant between ligand and receptor
K_L_	0.567	μM	Low affinity binding constant between ligand and receptor
K_C_	8.809	μM	affinity binding constant between free receptor and Gs
k_act1_	0.1	s^-1^	Activation rate constant for RGs complexes
k_act2_	5	s^-1^	Activation rate constant for LRGs complexes
k_hydr_	0.8	s^-1^	Hydrolization rate constant of G_Sα-GTP_
K_reas_	1.21*10^3^	s^-1^ μM ^-1^	Re-association rate constant of G_SαGDP_ and G_Sβγ_
G_sTotalCav_	10	μM	Concentration of G_s_ protein in Cav compartment
AC_5/6-Cav_	3.379	μM	Concentration of Cav AC_5/6_
ATP	5*10^3^	μM	Concentration of ATP
K_mATP_	315	μM	AC_5/6_ K_m_ for ATP
AF_5/6_	500	$\frac{mg\;purified\;protein}{mg\;membrane\;protein}$	Amplification factor for AC_5/6_
MW_AC5/6_	130	KD_a_	Molecular weight of AC_5/6_
PDE_2_	4.5	μM	PDE2 concentration in Cav compartment
k_PDE2_	20	s^-1^	Rate constant for PDE2
K_mPDE2_	50	μM	PDE2 Km for cAMP
PDE_3_	5.6	μM	PDE3 concentration in Cav compartment
k_PDE3_	1.25	s^-1^	Rate constant for PDE3
K_mPDE3_	0.08	μM	PDE3 Km for cAMP
PDE_4_	2.0	μM	PDE4 concentration in Cav compartment
k_PDE4_	2.5	s^-1^	Rate constant for PDE4
K_mPDE4_	2.2	μM	PDE4 Km for cAMP
L_iso_	30	nM	Isoproterenol concentration

**Table 2:** Initial values.

Variables	Values	Units
cAMP	0.1	μM
G_SαGTP_	0.042	μM
G_SαGDP_	0.0	μM
G_Sβγ_	0.042	μM

### Functional barrier model: 3-dimensional stochastic model and 1-dimensional continuum model

We also constructed a 3-dimensional stochastic model of cAMP diffusion that was implemented in MCell and visualized using CellBlender (mcell.org). The model consisted of a single caveolar domain (100 x 100 nm) flanked by extra-caveolar space for a total of 200 x 200 x 1000 nm. We define the z direction as orthogonal to the membrane. The boundary conditions were assumed to be no flux boundaries. The caveolar domain contained 15 β_1_ARs, which generated cAMP at 120 molecules/s. βARs and AC5/6 were placed in the plasma membrane associated with caveolar domains. cAMP freely diffused in space. MCell tracks diffusion in radial coordinates by dividing an octant of a sphere into 16384 directions (dphi ≈ 0.005 degrees). PDE molecules were placed in the plane z = L* = 100 nm as functional barriers. The Interaction radius is set to default [63–65]. The time step (Δ*t*) was set to 5.0 x 10^−9^ s so that $p_{b}=\frac{k∙\sigma }{2N_{a}}\sqrt{\frac{\pi ∙\Delta t}{D}}<1$, where *k* is binding rate, *σ* is surface grid density, *N*_*a*_ is the Avogadro constant, and *D* is diffusion [64]. σ is set according to the following table (Table 3):

**Table 3:** Values of surface grid density.

PDEs	Surface Grid Density *σ* (position/μm^2^)
10	1,500
100	2,500
1,000	25,000
10,000	250,000
100,000	2,500,000

The cAMP-PDE reaction was set to

$$cAMP+PDE\begin{array}{c} \overset{K_{f}}{\to }\\ \underset{K_{b}}{\leftarrow } \end{array}PDEcAMP\overset{K_{cat}}{\to }PDE$$ (14)

where *K*_*f*_ = 1.2 x 10^7^ M^-1^ s^-1^, *K*_*b*_ = 58.82 s^-1^, and *K*_*cat*_ = 14.70 s^-1^ [66].

Even with the smallest cAMP diffusion constant used in this study, the diffusion length for relevant time scales is much larger than the length scale of the caveolar/extracaveolar anatomical microstructure. Therefore, the distribution of cAMP in planes with fixed z are approximately uniform, and the distribution (effective concentration) of cAMP as a function of z can be approximated by a 1-dimensional continuum model. (See S1 Appendix for details). This model can be solved to obtain an expression for the steady state distribution of cAMP

$$cAMP\left(z\right)=\{\begin{array}{cc} (A+B)-\frac{J_{B}}{D}z, & \;0<z<L^{*}\\ B, & \;L^{*}<z<L \end{array},$$ (15)

where

$$A=\frac{J_{B}}{D}L^{*}\;\text{and}\;B=\frac{J_{B}}{K_{f}}\frac{K_{cat}+K_{b}}{L^{*}PDE_{0}K_{cat}\text{-}J_{B}},$$

Unless otherwise specified, the rate constants *K*_*f*_, *K*_*b*_, and *K*_*cat*_ are as defined above, the flux of cAMP into the domain is *J*_*B*_ = 4.982 μm μM s^-1^, and the location of the functional barrier is L* = 100 nm. *PDE*_*tot*_ is the total concentration of bound and unbound PDE and is taken to be 4.1514 x 10^n^ μM for n = 0,1,2,3, and 4, where the concentration is averaged over the region from the plasma membrane at z = 0 to the PDE barrier at z = L*. (Note that the product of *PDE*_*tot*_ and the cross-sectional area of the microdomain and L* is the total number of PDE molecules.)

### 3-dimensional continuum anatomical barrier model of the dyadic junction

For the models that included a physiological geometry, this geometry was developed from cryo-TEM images of adult mouse cardiac myocytes, as described previously [45] (see Fig 7). Briefly, a tetrahedral surface mesh was imported into Blender for finite element simulations and to smooth the sharp edges from segmentation of the TEM images (using Blamer) that would impede numerical modeling and lead to artifacts. BLAMer is a plug-in for the Open Source Blender visualization environment (http://www.blender.org) that provides an interactive interface to the GAMer (Geometry-preserving Adaptive Mesher) tool (http://nbcr.ucsd.edu/?page_id=1131) from the FEtk (Finite Element ToolKit) software package (http://nbcr.ucsd.edu/?page_id=495) maintained and distributed by the NIH-supported National Biomedical Computation Resource. GAMer produces high-quality simplex meshes of surfaces and volumes and was used via BLAMer by Hake et al. [45] to mesh the myocyte dyadic cleft anatomy from 3D electron tomographic data. This mesh was resegmented and imported into Virtual Cell. This mesh included two t-tubules surrounded by SR as well as two mitochondria.

## Supporting Information

S1 Fig Idealized PDE model demonstrating cAMP generation and diffusion from t-tubular caveolar microdomains (seen as rectangles along edge of inset) at various time points following β_1_AR stimulation with 30 nM isoproterenol (basal cAMP = 0.1 μM). As expected, in the presence physiological concentrations of PDEs, small gradients were shown in panel (A) The diffusion coefficient is 60 μm^2^/s. (B) The diffusion coefficient is 10 μm^2^/s. In the presence of physiological concentrations of PDEs, diffusion is fast and gradients very small.
(TIF)

S2 Fig Using experimental observed cAMP concentrations as initial values in bulk (1.0 μM) and caveolar (0.1 μM) microdomains. **Simulated cAMP concentration diffusion in 2-D continuum model without adenylyl cyclase stimulations in the caveolar microdomain with 10-fold increase in the concentration of PDEs.** Left panel in (A) shows the cAMP in Cav reached 1.0 μM in 0.001 seconds with diffusion = 300 μm^2^/s. In the longer time interval shown in the right panel, cAMP concentrations in both microdomains declined to 0.1 μM. Right panels show longer simulation in time, with reduction in cAMP due to PDE digestion. The purple boxes in the right panels indicate the time interval shown in left panels. (B) The diffusion rate is 60 μm^2^/s. In this case, cAMP concentration in Cav reached 1.0 μM within 0.003 seconds. (C) With a slower diffusion rate (10 μm^2^/s), cAMP instantly reached 0.9645 μM in the Cav domain and then declined to 0.1 μM in the bulk and Cav in 2.5 seconds.
(TIF)

S3 Fig Stochastic simulation of cAMP diffusion implemented in MCell and visualized using CellBlender. The diffusion coefficient was set to ***60 μm***^***2***^***/s***. (A) 10 PDE molecules (~4.1514 μM). Four snapshots are shown in the left, the average cAMP concentration for the 1800 time frames between 1s to 10s are shown in the blue bar graph, and the time course of the spatially averaged cAMP concentration is shown in the top right panel. (B) PDE molecules = 100 (~41.514 μM). Average of cAMP molecules for 1800 time frames from 1s to 10s at steady state. (C) PDE molecules = 1000 (~415.14μM). Average of cAMP molecules over 1800 time frames from 1s to 10s at steady state. (D) PDE molecules = 10000 (~4151.4 μM). Average of cAMP molecules for 1800 time frames from 1s to 10s at steady state. (E) PDE molecules = 100000 (~41514 μM). Average of cAMP molecules over 1800 time frames from 1s to 10s at steady state. The red curves plotted on the accumulated concentration maps in panel (B-E) show the predictions of the 1D continuum model. In all cases, there is excellent agreement with the full 3D stochastic model. The cAMP compartmentation ratio *R* for the various values of PDE concentration shown in panels (A-E) are 3.048 x 10^−4^, 1.502 x 10^−2^, 1.414 x 10^−1^, 6.239 x 10^−1^, and 9.432 x 10^−1^.
(TIF)

S1 Appendix 1-Dimensional continuum functional barrier model. (DOCX)

S2 Appendix Codes using CellBlender for 3-dimensional stochastic model. (ZIP)
