# Supplementary figures and images for: A Computational Modeling and Simulation Approach to Investigate Mechanisms of Subcellular cAMP Compartmentation
Source: PLoS Comput Biol. 2016 Jul 13;12(7):e1005005. doi: 10.1371/journal.pcbi.1005005 (PMC4943723; doi:10.1371/journal.pcbi.1005005)

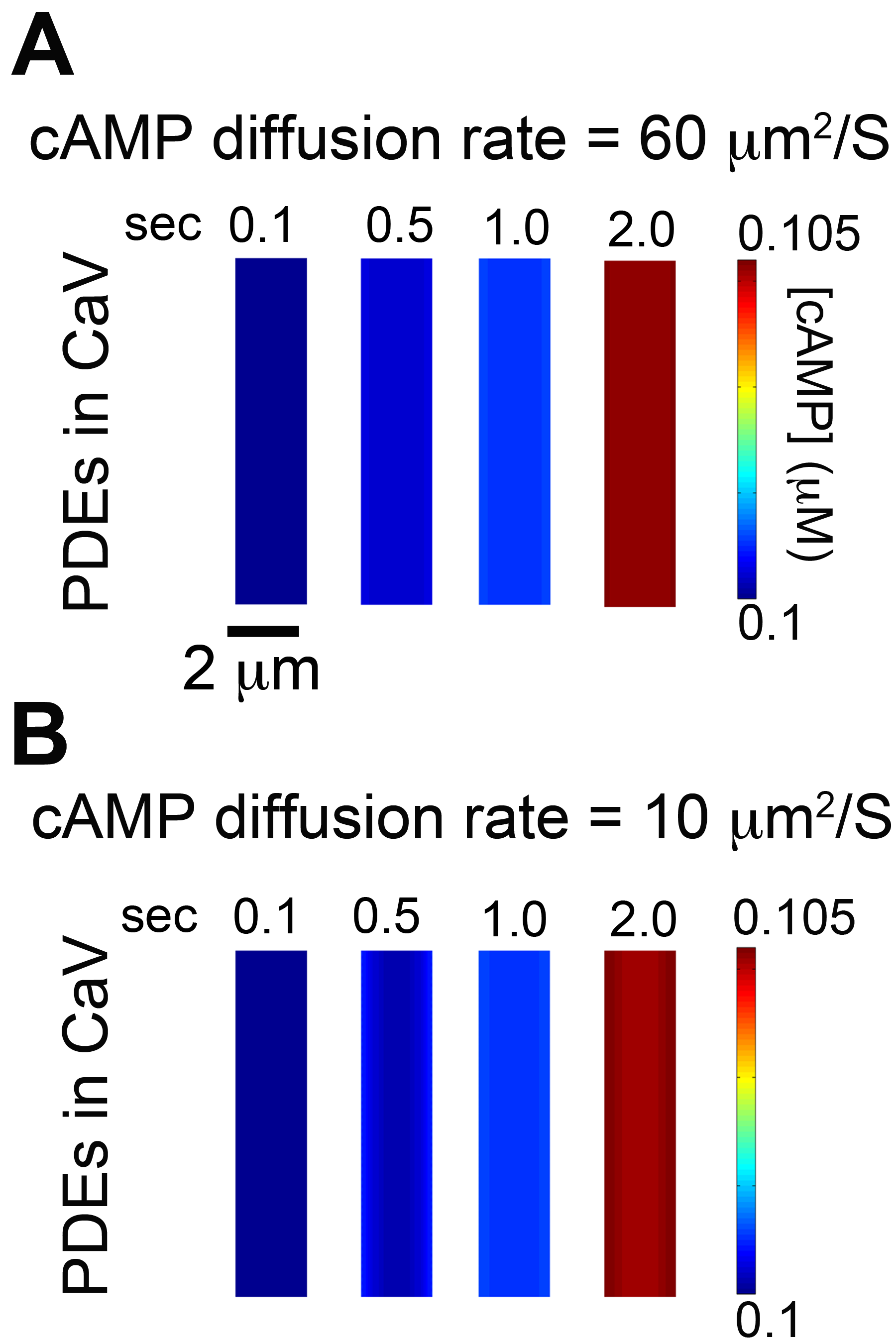

Supplement: S1 Fig — As expected, in the presence physiological concentrations of PDEs, small gradients were shown in panel (A) The diffusion coefficient is 60 μm2/s. (B) The diffusion coefficient is 10 μm2/s. In the presence of physiological concentrations of PDEs, diffusion is fast and gradients very small. (TIF) [file pcbi.1005005.s001.tif]

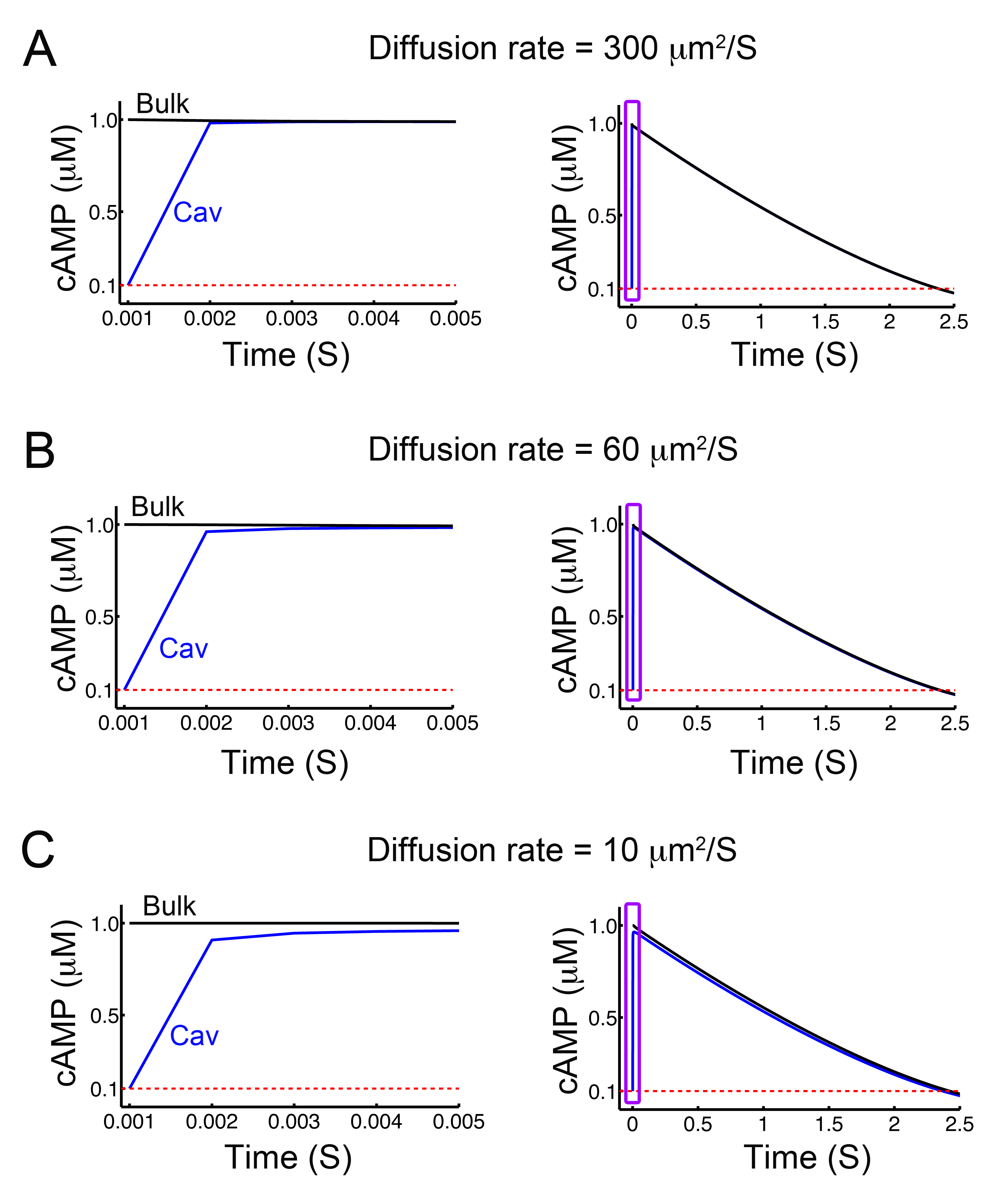

Supplement: S2 Fig — Simulated cAMP concentration diffusion in 2-D continuum model without adenylyl cyclase stimulations in the caveolar microdomain with 10-fold increase in the concentration of PDEs. Left panel in (A) shows the cAMP in Cav reached 1.0 μM in 0.001 seconds with diffusion = 300 μm2/s. In the longer time interval shown in the right panel, cAMP concentrations in both microdomains declined to 0.1 μM. Right panels show longer simulation in time, with reduction in cAMP due to PDE digestion. The purple boxes in the right panels indicate the time interval shown in left panels. (B) The diffusion rate is 60 μm2/s. In this case, cAMP concentration in Cav reached 1.0 μM within 0.003 seconds. (C) With a slower diffusion rate (10 μm2/s), cAMP instantly reached 0.9645 μM in the Cav domain and then declined to 0.1 μM in the bulk and Cav in 2.5 seconds. (TIF) [file pcbi.1005005.s002.tif]

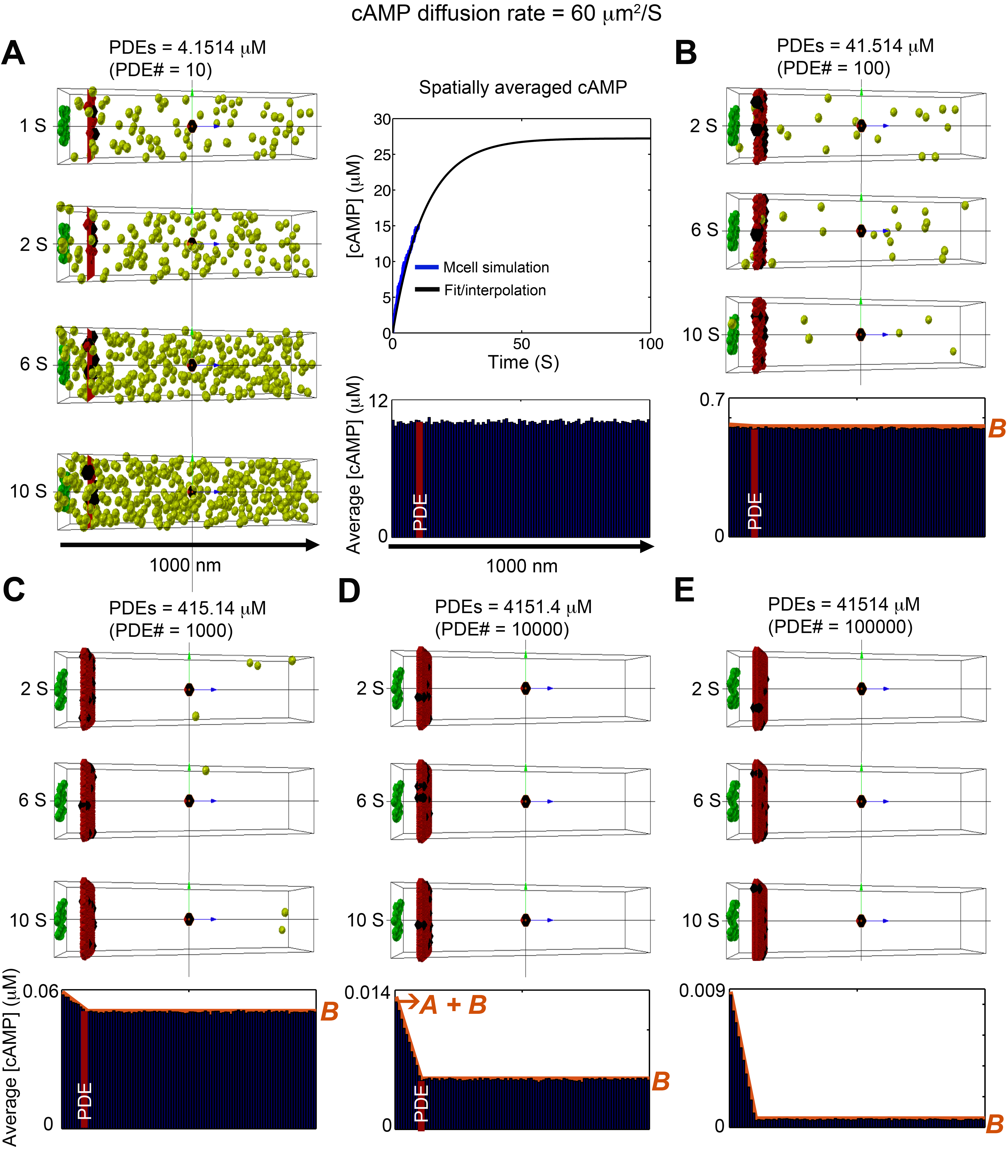

Supplement: S3 Fig — The diffusion coefficient was set to 60 μm2/s. (A) 10 PDE molecules (~4.1514 μM). Four snapshots are shown in the left, the average cAMP concentration for the 1800 time frames between 1s to 10s are shown in the blue bar graph, and the time course of the spatially averaged cAMP concentration is shown in the top right panel. (B) PDE molecules = 100 (~41.514 μM). Average of cAMP molecules for 1800 time frames from 1s to 10s at steady state. (C) PDE molecules = 1000 (~415.14μM). Average of cAMP molecules over 1800 time frames from 1s to 10s at steady state. (D) PDE molecules = 10000 (~4151.4 μM). Average of cAMP molecules for 1800 time frames from 1s to 10s at steady state. (E) PDE molecules = 100000 (~41514 μM). Average of cAMP molecules over 1800 time frames from 1s to 10s at steady state. The red curves plotted on the accumulated concentration maps in panel (B-E) show the predictions of the 1D continuum model. In all cases, there is excellent agreement with the full 3D stochastic model. The cAMP compartmentation ratio R for the various values of PDE concentration shown in panels (A-E) are 3.048 x 10^−4^, 1.502 x 10^−2^, 1.414 x 10^−1^, 6.239 x 10^−1^, and 9.432 x 10^−1^. (TIF) [file pcbi.1005005.s003.tif]
