# Supplementary material for: A Computational Modeling and Simulation Approach to Investigate Mechanisms of Subcellular cAMP Compartmentation
Source: PLoS Comput Biol. 2016 Jul 13;12(7):e1005005. doi: 10.1371/journal.pcbi.1005005 (PMC4943723; doi:10.1371/journal.pcbi.1005005)
Supplement: S1 Appendix — (DOCX) [file pcbi.1005005.s004.docx]

***S1 APPENDIX: 1-Dimensional Continuum Functional Barrier Model***

In this appendix, we derive the 1-dimensional (1D) continuum functional barrier model and provide an analytical solution for the steady state concentration of cAMP.

Our 3-dimensional stochastic functional barrier model of cAMP diffusion consisted of a single caveolar domain ($100 \times100 \mathrm{nm}$) flanked by extra-caveolar a space for a total of $200 \times200 \times1000$nm. (See figure 1D). cAMP freely diffused in space. βARs and AC5/6 were placed in the plasma membrane associated with caveolar domains and generated a flux of cAMP into the domain. Otherwise boundary conditions were taken to be no flux. PDE molecules were placed in the plane $z=L^{*}$ as a functional barrier, where the $z$ direction was defined to be orthogonal to the membrane. PDE and cAMP molecules could react if the distance between them was less than an "interaction radius" of $\varepsilon/2$. The reaction kinetics between cAMP and PDE were given by

$$cAMP+PDE\begin{matrix} \underset{\to}{K_{f}} \\ \overset{\leftarrow}{K_{b}} \end{matrix}PDEcAMP\underset{\to}{K_{cat}}PDE , \ldots\left( a1 \right),$$

where $K_{f}=12.0 {\mu M}^{-1}s^{-1}$, $K_{b}=58.82 s^{-1}$, and $K_{cat}=14.70 s^{-1}$ ^34^.

Even with the smallest cAMP diffusion coefficient considered in this manuscript (10 μm^2^/s), the diffusion length (2$\sqrt{\mathrm{Dt}}$) of cAMP on relevant time scales (1-10 seconds) is much larger than the length scale of the cross-sectional area of the microdomain domain ($\Delta x=\Delta y=200 \mathrm{nm}$) and the cAMP-PDE interaction radius $\varepsilon/2$. This leads to a nearly uniform concentration of cAMP in planes parallel to the plasma membrane along the microdomain (fixed $z$), and therefore the effective concentration of cAMP as a function of $z$ can be approximated by a 1D continuum model.

In the 1D continuum model, cAMP dynamics within the microdomain are described by the diffusion equation

$$\frac{\partial}{\partial t}\left( cAMP\left( z,t \right) \right)=D\frac{\partial^{2}}{{\partial z}^{2}}\left( cAMP\left( z,t \right) \right)+\eta_{\varepsilon}\left( z-L^{*} \right) f\left( cAMP\left( z,t \right), PDE(t) \right) ,$$

where $cAMP\left( z,t \right)$ is the concentration of cAMP (in $\mu M$), $D$ is the diffusion constant of cAMP (in ${\mu m}^{2}/s$), $L$ is the length of the microdomain ($1000 \mathrm{nm}$), $t$ is time (in $s$), and $z$ is the distance along the microdomain (in $\mu m$). The reaction term $\eta_{\varepsilon}\left( z-L^{*} \right) f\left( cAMP\left( z,t \right), PDE(t) \right)$ accounts for the effects of the PDE barrier. This reaction term is localized to a barrier region centered at the position of the PDE molecules ($z=L^{*}$) and has a thickness of twice the cAMP-PDE interaction radius ($\varepsilon$), i.e.,

$$\eta_{\varepsilon}\left( z-L^{*} \right)=\left\{ \begin{matrix} 1, & L^{*}-\frac{\varepsilon}{2}<z<L^{*}+\frac{\varepsilon}{2} \\ 0, & elsewhere \end{matrix} \right. .$$

$PDE\left( t \right)=\frac{nPDE\left( t \right)}{\varepsilon\Delta x \Delta y}$ is the effective concentration of PDE (in $\mu M$) in the reaction region, i.e., the number of unbound PDE molecules $nPDE\left( t \right)$ divided by the volume of the PDE-cAMP interaction region. Mass action kinetics for reaction scheme $\left( a1 \right)$ provide the magnitude of reaction term is given by the

$$f\left( cAMP, PDE \right)={-K}_{f} cAMP PDE+ K_{b}\left( {PDE}_{tot}-PDE \right),$$

where ${PDE}_{tot}=\frac{{nPDE}_{tot}}{\varepsilon A}$ is the total concentration of PDE (bound or unbound) in the reaction region, and the dynamics of the PDE

$$\frac{dPDE}{dt}=-K_{f} {cAMP}_{b} PDE+\left( K_{b}+K_{cat} \right)\left( {PDE}_{tot}-PDE \right),$$

where ${cAMP}_{b}={cAMP}_{b}\left( t \right)$ is the average cAMP concentration within the barrier region

$${cAMP}_{b}\left( t \right)=\frac{1}{\varepsilon} \int_{L^{*}-\frac{\varepsilon}{2}}^{L^{*}+\frac{\varepsilon}{2}} cAMP\left( z,t \right) dz.$$

To complete the 1D continuum model, the boundary conditions are set to account for the influx of cAMP at the plasma membrane ($z=0$) and to correspond to zero flux at the cytosolic end of the microdomain ($z=L)$

$$-D\left. \frac{\partial cAMP}{\partial z} \right|_{z=0}=J_{B}, -D\left. \frac{\partial cAMP}{\partial z} \right|_{z=L}=0 ,$$

where $J_{B}$ is the flux of cAMP into the microdomain due to βAR activity ($4.982 \mu m \mu M/s$),

Because the interaction radius $\varepsilon/2$ is much smaller than the diffusion length, the thickness of the PDE barrier can be reduced to a point at $z=L^{*}$ in a manner that preserves the strength of effect of the reaction. That is, $\eta_{\varepsilon}\left( z-L^{*} \right)$ can be well approximated by a delta-function $\varepsilon\delta\left( z-L^{*} \right)$ and ${cAMP}_{b}\left( t \right)\approx cAMP\left( L^{*},t \right)$. Therefore, the full 1D functional barrier model is

$$\left\{ \begin{matrix} \begin{matrix} \begin{matrix} \frac{\partial cAMP}{\partial t}=D\frac{\partial^{2}cAMP}{{\partial z}^{2}}+ \delta\left( z-L^{*} \right) \varepsilon\left( -K_{f} cAMP PDE+ K_{b}\left( {PDE}_{tot}-PDE \right) \right) \\ \end{matrix} \\ \\ \end{matrix} \\ \begin{matrix} \frac{dPDE}{dt}=-K_{f} cAMP\left( L^{*},t \right) PDE+ \left( K_{b}+K_{cat} \right)\left( {PDE}_{tot}-PDE \right) \\ \\ \end{matrix} \\ -D\left. \frac{\partial cAMP}{\partial z} \right|_{z=0}=J_{B}, -D\left. \frac{\partial cAMP}{\partial z} \right|_{z=L}=0 \end{matrix} \right. , \ldots(a2).$$

Setting $\frac{\partial cAMP}{\partial t}=0, \frac{\partial PDE}{\partial t}=0$ in the system $(a2)$, we obtain equations for the steady state concentration of cAMP along the microdomain, $cAMP\left( z \right)$, and the steady state effective concentration of PDE at the barrier, ${PDE}^{*}$. The presence of the delta-function at $z=L^{*}$ in the reaction term requires that we solve the system on $0<z<L^{*}$ and $L^{*}<z<L$, and then apply the boundary conditions and appropriately match the solutions in these two regions at $z=L^{*}$.

(i) Within the microdomain with $z\neq L^{*}$, the steady state equation for cAMP concentration $cAMP\left( z \right)$ is

$$D\frac{\partial^{2}cAMP}{{\partial z}^{2}}=0,$$

and therefore

$$cAMP\left( z \right)={cAMP}_{1}\left( z \right)=A_{1}\left( z-L^{*} \right)+B_{1}, 0<z<L^{*},$$

$$cAMP\left( z \right)={cAMP}_{2}\left( z \right)=A_{2}\left( z-L^{*} \right)+B_{2}, L^{*}<z<L,$$

for some constants $A_{1}{, A}_{2},B_{1},$and $B_{2}$.

(ii) The boundary conditions $-D\left. \frac{\partial{cAMP}_{1}}{\partial z} \right|_{z=0}=J_{B}$ and $-D\left. \frac{\partial{cAMP}_{2}}{\partial z} \right|_{z=L}=0$ imply that $A_{1}=-\frac{J_{B}}{D}$ and $A_{2}=0$, respectively.

(iii) Because the cAMP concentration $cAMP\left( z \right)$ must be continuous at all $z$ including $z=L^{*}$, $B_{1}=B_{2}=cAMP\left( L^{*} \right)$.

(iv) The difference in the flux of cAMP into and out of $z=L^{*}$ must be balanced by the effects of the PDE-cAMP reaction,

$$-D\left. \frac{\partial{cAMP}_{2}}{\partial z} \right|_{z=L^{*}}+D\left. \frac{\partial{cAMP}_{1}}{\partial z} \right|_{z=L^{*}}=\varepsilon\left( -K_{f} cAMP\left( L^{*} \right) {PDE}^{*}+ K_{b}\left( {PDE}_{tot}-{PDE}^{*} \right) \right)$$

(Note the this condition can be obtained by integrating the steady state differential equation for cAMP across the delta-function)$.$ Substituting the information from (i)-(iii) into this expression yields

$$- J_{B}=\varepsilon\left( -K_{f} cAMP\left( L^{*} \right) {PDE}^{*}+ K_{b}\left( {PDE}_{tot}-{PDE}^{*} \right) \right) \ldots(a3).$$

(v) Finally, the steady state equation for the concentration of unbound PDE ${PDE}^{*}$ is

$$0=-K_{f} cAMP\left( L^{*} \right) {PDE}^{*}+ \left( K_{b}+K_{cat} \right)\left( {PDE}_{tot}-{PDE}^{*} \right) \ldots(a4).$$

We obtain $cAMP\left( L^{*} \right)$ and ${PDE}^{*}$ by solving equations $(a3)$ and $(a4)$

$$cAMP\left( L^{*} \right)=\frac{J_{B}}{K_{f}} \frac{\left( 1+\frac{K_{b}}{K_{cat}} \right)}{\left( \varepsilon{PDE}_{tot}-\frac{J_{B}}{K_{cat}} \right)} ,$$

and

$${PDE}^{*}={PDE}_{tot}-\frac{J_{B}}{\varepsilon K_{cat}} .$$

Therefore,

$$cAMP\left( z \right)=\left\{ \begin{matrix} \begin{matrix} -\frac{J_{B}}{D}z+A+B , 0\leq z<L^{*} \\ \end{matrix} \\ B , L^{*}\leq z\leq L \end{matrix} \right. ,$$

where

$$A= \frac{J_{B}}{D}L^{*} ,$$

and

$$B= \frac{J_{B}}{K_{f}} \frac{\left( 1+\frac{K_{b}}{K_{cat}} \right)}{\left( \varepsilon{PDE}_{tot}-\frac{J_{B}}{K_{cat}} \right)} .$$

This implies that there is a linear decay in cAMP concentration from the plasma membrane ($z=0$) to the PDE barrier at $z=L^{*}$ and is constant beyond the barrier.

Note that the effective concentration of PDE in the above expression is taken with respect to the width of the cAMP-PDE interaction region $\varepsilon$. In the main manuscript, despite all of the PDE molecules being located at $z=L^{*}$, the effective concentration of PDE is taken with respect to the region between the plasma membrane and the PDE barrier at at $z=L^{*}$. Therefore, the expression $cAMP\left( z \right)$ in the main manuscript has $L^{*}$ in place of $\varepsilon$.

The compartmentation ratio (as described in the main manuscript is

$$R = \frac{A}{A+B} = \frac{1}{1+\frac{D}{{L^{*} K}_{f}}\left( \frac{K_{cat}+K_{b}}{\varepsilon{PDE}_{tot}K_{cat}-J_{B}} \right)} .$$

This expression reveals that

(i) As the cAMP production rate $J_{B}$ increases towards a critical value ${J_{B}}^{*}=\varepsilon{PDE}_{tot}K_{cat}$, the compartmentation approaches 0, i.e., no compartmentation. For $J_{B}{{\geq J}_{B}}^{*}$, PDE is saturated and cAMP grows unboundedly, i.e., there is no steady state.

(ii) For $0<J_{B}{{<J}_{B}}^{*}$, the compartmentation ratio $R$ increases as the cAMP production rate $J_{B}$ decreases. In the limit of $J_{B}$ going to 0, $R$ approaches

$$R_{0} = \frac{1}{1+\frac{D}{{L^{*} K}_{f}}\left( \frac{K_{cat}+K_{b}}{\varepsilon{PDE}_{tot}K_{cat}} \right)} ,$$

which provides an *upper limit* to compartmentation. For all parameter sets use in the manuscript, $J_{B}$ is small compared ${J_{B}}^{*}$ except at small PDE concentrations, and plots of $R$ vs. ${PDE}_{tot}$ are nearly indistinguishable from those of $R_{0}$ vs. ${PDE}_{tot}$ on the scale used in Figure 5. This agrees with tOK very low sensitivity in response to changes in $J_{B}$ reported in the main text.
